# Intracranial pressure elevation alters CSF clearance pathways

**DOI:** 10.1186/s12987-020-00189-1

**Published:** 2020-04-16

**Authors:** Vegard Vinje, Anders Eklund, Kent-Andre Mardal, Marie E. Rognes, Karen-Helene Støverud

**Affiliations:** 1 Department of Scientific Computing and Numerical Analysis, Simula Research Laboratory, Lysaker, Norway; 2 Department of Radiation Sciences, Umeå University, Umeå, Sweden; 3 Department of Mathematics, University of Oslo, Oslo, Norway

**Keywords:** Infusion test, CSF circulation, Glymphatic pathway, CSF dynamics, Intracranial pressure, Paravascular flow

## Abstract

**Background:**

Infusion testing is a common procedure to determine whether shunting will be beneficial in patients with normal pressure hydrocephalus. The method has a well-developed theoretical foundation and corresponding mathematical models that describe the CSF circulation from the choroid plexus to the arachnoid granulations. Here, we investigate to what extent the proposed glymphatic or paravascular pathway (or similar pathways) modifies the results of the traditional mathematical models.

**Methods:**

We used a compartment model to estimate pressure in the subarachnoid space and the paravascular spaces. For the arachnoid granulations, the cribriform plate and the glymphatic circulation, resistances were calculated and used to estimate pressure and flow before and during an infusion test. Finally, different variations to the model were tested to evaluate the sensitivity of selected parameters.

**Results:**

At baseline intracranial pressure (ICP), we found a very small paravascular flow directed into the subarachnoid space, while 60% of the fluid left through the arachnoid granulations and 40% left through the cribriform plate. However, during the infusion, 80% of the fluid left through the arachnoid granulations, 20% through the cribriform plate and flow in the PVS was stagnant. Resistance through the glymphatic system was computed to be 2.73 mmHg/(mL/min), considerably lower than other fluid pathways, giving non-realistic ICP during infusion if combined with a lymphatic drainage route.

**Conclusions:**

The relative distribution of CSF flow to different clearance pathways depends on ICP, with the arachnoid granulations as the main contributor to outflow. As such, ICP increase is an important factor that should be addressed when determining the pathways of injected substances in the subarachnoid space. Our results suggest that the glymphatic resistance is too high to allow for pressure driven flow by arterial pulsations and at the same time too small to allow for a direct drainage route from PVS to cervical lymphatics.

## Background

Infusion testing is a standard procedure to assess whether patients with normal pressure hydrocephalus (a type of dementia) would benefit from shunt surgery. During infusion of artificial cerebrospinal fluid (CSF), intracranial pressure (ICP) is monitored, and a CSF outflow resistance ($R_{\text {out}}$) is calculated. Typically, a constant infusion rate of 1.5 mL/min results in an ICP increase by around 10–25 mmHg, and the calculated $R_{\text {out}}$ parameter is commonly used as a supplementary parameter in the selection of patients for shunt surgery [1]. The procedure has a well developed theoretical foundation as well as corresponding mathematical models (see [2] for an overview). The main outflow route is assumed to be the arachnoid granulations (AG) [3] in accordance with the traditional view of the third circulation where CSF is produced in the choroid plexus and absorbed through AG as described by Cushing in 1925 [4].

More recently, an alternative CSF circulation has been proposed—the glymphatic circulation. Here, paravascular spaces (PVS), extensions of the Virchow–Robin spaces, play an active role in a brain-wide CSF circulation in conduits that run in parallel with the vasculature. The purpose of this circulation is to clear solutes from deep inside the brain, thus taking the role of the lymphatic system within the central nervous system which is absence of lymphatic vessels. Therefore, this waste clearance system has been named the glymphatic system [5], where the “g” indicates that glial cells play an important role. Glymphatic dysfunction has been hypothesized to contribute to development in neurodegenerative disorders, traumatic brain injury and stroke [5]. In the glymphatic circulation, CSF moves through the subarachnoid space (SAS) along arteries and dives into the brain along arterial PVS. The glymphatic pathway enters the extracellular space (ECS) through AQP-4 channels or inter-endfeet gaps, and from there eventually reaches the venous PVS.

Most of the evidence for the glymphatic pathway has been established via in-vivo rodent experiments. In these experiments, tracers are typically infused in the CSF in rodents at a rate of $0.34{-}2 \, \upmu \text {L/min}$, with a resulting pressure increase of 0.1–2.5 mmHg [6–8]. Even though CSF turnover time differs between mice and men [9], an infusion rate of 1.5 mL/min and a total CSF volume up to 350 mL in humans [10], and in some cases possibly as low as 100 mL [9] is comparable to an infusion rate of $\approx 0.15 \, \upmu \text {L/min}$ and a total CSF volume of $35 \, \upmu \text {L}$ in mice [9]. Thus, such tracer experiments may in fact be viewed as infusion tests. This potential link, between infusion tests and the glymphatic system, has not yet been explored.

Recently, the resistance of the glymphatic system under normal conditions was estimated by Faghih and Sharp [11]. They concluded that the glymphatic circulation was unlikely, as the high resistance of the pathway would prevent sufficient flow. In their model, the resistance of the paraarterial tree was relatively low before reaching the precapillary level where the gap size of the PVS was set to 100 nm in accordance with a study of Bedussi et al. [7]. The narrow PVS at the capillary level effectively blocked the circulation. However, other studies suggest flow within the paravascular spaces at the level of capillaries [12, 13]. Furthermore, it has been argued that fixation, which was used by Bedussi et al. [7], shrinks the PVS [8]. As such, the resistance of the PVS at the capillary level should be further investigated and compared to the low permeability in the ECS of the brain parenchyma [14–16].

There is also compelling evidence of flow directly from the SAS to the lymphatic system. In earlier works, Bradbury et al. [17] reported that at least 30% of CSF drains to cervical lymphatics. More recently, Ma et al. suggest that lymphatic outflow is responsible for the main portion of CSF leaving the SAS [18], and that flow through the cribriform plate dominates the paravascular flow route when total CSF efflux is large [19]. In sheep it has been reported that outflow through the cribriform plate plays a major role in CSF absorption, whereas the importance of the AG is unclear [20].

Within the brain parenchyma, the Bulat-Klarica-Orešković hypothesis states that production and absorption of CSF mainly occurs over the capillary wall due to its large surface area [21]. Other CSF outflow routes have also been proposed [22, 23], however, a quantification of the fluid distribution and interplay between each outflow pathway is yet to be properly addressed. In particular, resistance of flow from the paraarterial space through the ECS and/or along capillaries in the setting of infusion tests (i.e. under temporarily elevated pressure) has not yet been investigated. We note that lumbar intrathecal contrast delivery during infusion to assess glymphatic function in humans was proposed in Yang et al. [24]. Further, as suggested by Ma et al. [19] increased flow, and a possible change in ICP, may alter the distribution of CSF to different outflow pathways. In addition, if the glymphatic circulation is a main outflow route for CSF, the outflow resistance $R_{\text {out}}$ is a direct measure of glymphatic dysfunction, which in turn has been linked to neurodegenerative disorders [5].

On this background, the aim of this work was to quantify different CSF outflow routes in the setting of an infusion test in supine position. To do so, we first gathered and summarized available resistances of the more probable outflow pathways including the AG, the cribriform plate, the arterial and venous PVS, and CSF drainage/filtration over the capillary wall. We next estimated resistances in the missing segments, i.e. the capillary gaps, the inter-endfeet (IEG) and the ECS. With these resistances, we extended a well-established mathematical infusion model to include additional pathways and then assessed the relative importance of the different outflow routes at baseline ICP and during infusion of fluid into the CSF system. We modeled an infusion test to explore potential changes in CSF outflow routes with the rising pressure. The relative importance of each outflow route was found to change with increasing ICP, although clearance through AG was dominant both at baseline and (elevated) plateau ICP. At baseline, flow in PVS was slow, with an average velocity of $0.18 \, \upmu \text {m/s}$ from the PVS into the SAS due to capillary filtration. During the infusion, PVS velocities decreased, and eventually stagnated.

## Methods

### Exit routes from the SAS

Mathematical models of the infusion test have usually assumed the AG to be the main exit route from the SAS [3, 25–28]. Our purpose here was to investigate and quantify the plausibility of other routes, in particular in light of the proposed glymphatic system [5, 29]. It should be noted that this is our interpretation of the glymphatic system on the macroscale, while the original hypothesis included both micro- and macroscopic processes. More precisely, we assumed three exit routes for CSF leaving the SAS, namely the AG entering the dural sinus, the cribriform plate (crib) entering into lymphatic vessels, and PVS [23]. From PVS, we assumed two further possible pathways: flow into the ECS through IEG, or flow along small gaps around capillaries [12, 13, 30, 31]. To reach the paravenous spaces, fluid from the ECS exits through IEG on the venous side, while the capillary gaps form a continuous space with the same paravenous spaces. From the paravenous spaces, the glymphatic circulation suggests a re-entry into the SAS or flow along paravenous spaces, possibly surrounded by pial sleeves [32], leading directly to cervical lymph [5, 29]. Although compelling evidence for direct contact between paravenous spaces and lymphatic vessels is lacking, such a route has been hypothesized by some investigators [33]. In this paper we assumed direct communication between PVS and SAS on both ends of the glymphatic system, and in a variation of our model, we tested whether a lymphatic exit route directly from venous PVS was plausible.

**Fig. 1:**
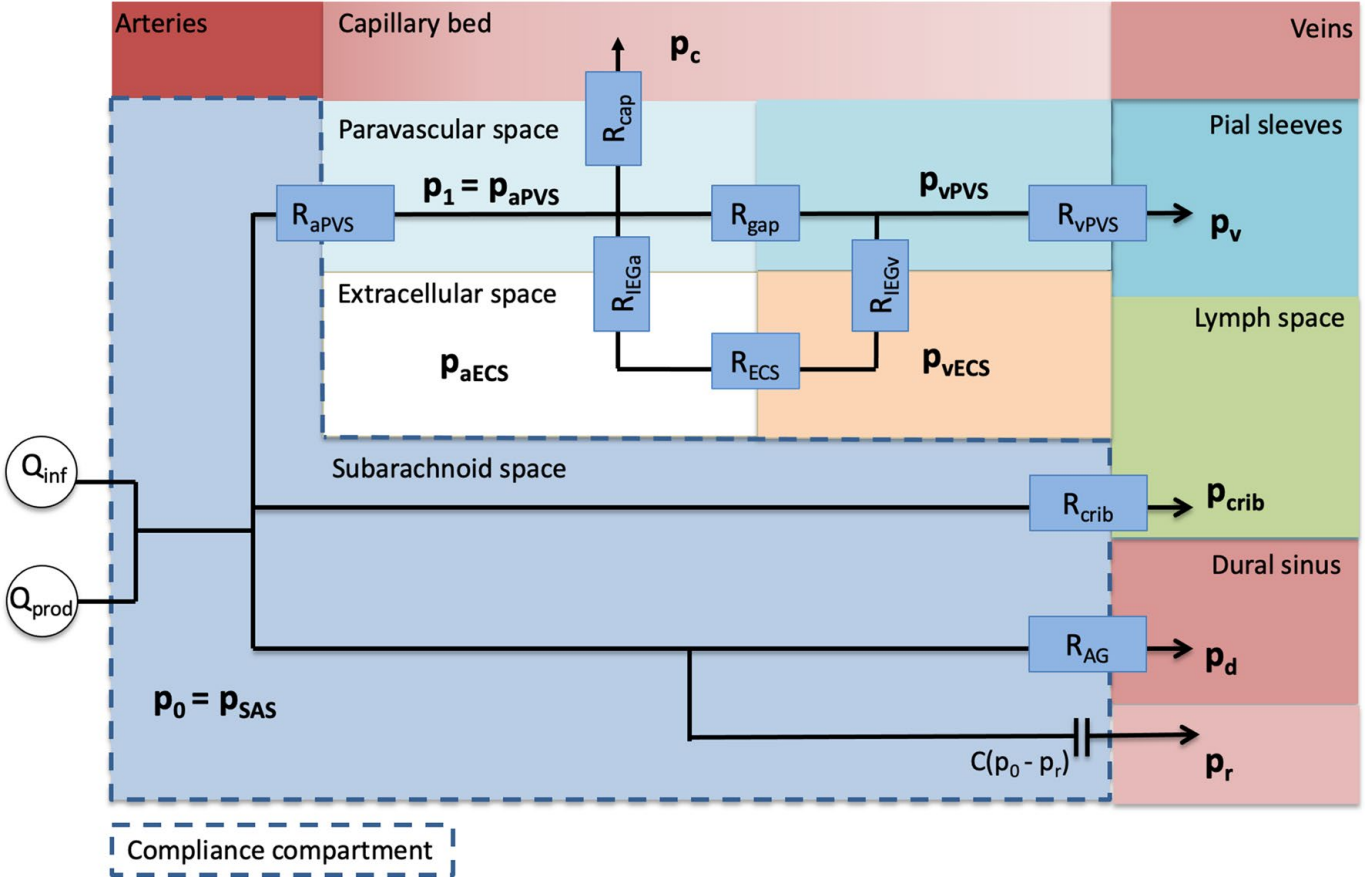
Schematic model description. The model relates the unknown $p_0 = p_{\text {SAS}}$ and $p_1 = p_{\text {PVS}}$ and three main exit pathways. CSF formed by production (${\text {Q}}_{{\text {prod}}}$) and infused fluid (${\text {Q}}_{{\text {inf}}}$) enter the SAS from the left. The first outflow route is via the arachnoid granulations (AG) where CSF is absorbed by the dural sinuses. The second route is via the cribriform plate (crib), where CSF is absorbed by extracranial lymphatic vessels. In the third outflow route, CSF enters the arterial paravascular spaces (aPVS), the fluid continues along gaps surrounding the capillaries (gaps) or enter the extracellular space (ECS) via the arterial inter-endfeet gaps (aIEG), before entering the venous paravascular spaces (vPVS) via the venous inter-endfeet gaps (vIEG), where the fluid is assumed to return to the SAS. The model also include filtration from the capillaries related to the effective capillary pressure ($p_{\text {e}}$)and in a variation of the model fluid flows directly from vPVS to cervical lympathics via pial sleeves ($p_v$). The SAS is considered as one pressure dependent compliance compartment (*C*), which is related to the reference pressure ($p_{\text {r}}$)

**Fig. 2:**
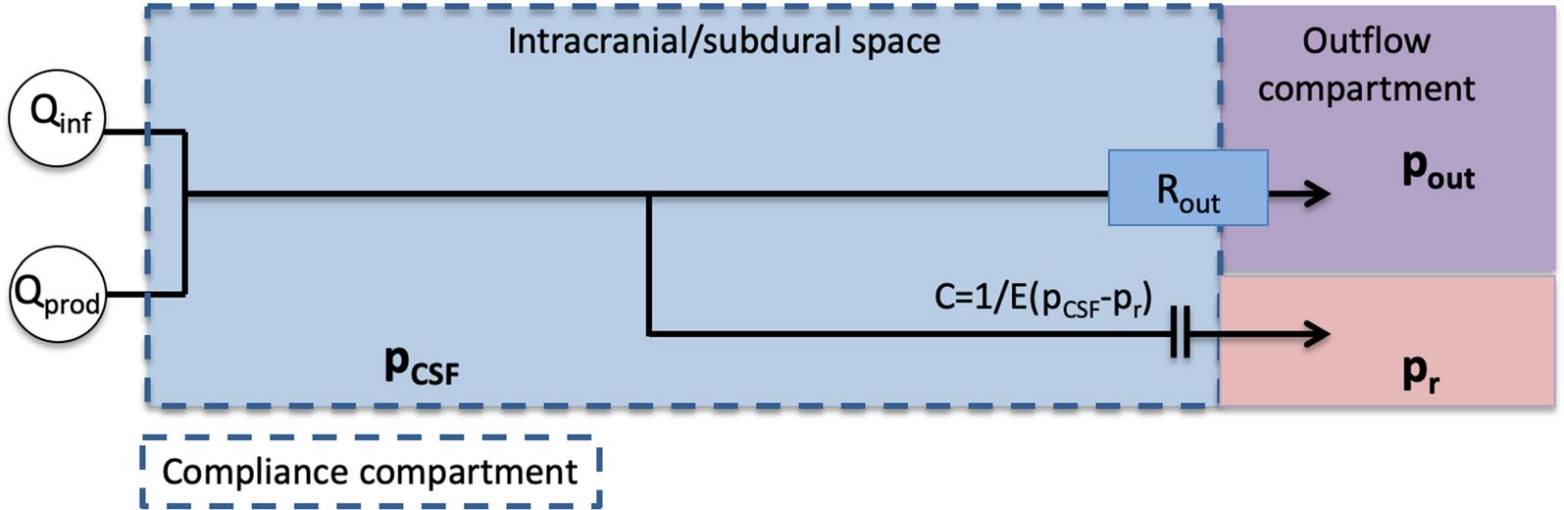
Reference model. An established model for analysis of clinical infusion tests, in which CSF flows out through the arachnoid granulations, with the dural sinus as the outflow compartment and $p_{\text {out}} = p_{\text {d}}$. The intracranial resting pressure (${\text {ICP}}_r$) is assumed to be related to the unknown $p_{{\text {out}}}$, $Q_{{\text {prod}}}$, and $R_{\text {out}}$ by $p_{\text {out}} = {\text {ICP}}_r - Q_{{\text {prod}}} R_{\text {out}}$

Figure 1 shows the full model, as an extension of the model often used in the literature assuming outflow through the AG only [27, 28]. Throughout, we will compare results from the full model, as shown in Fig. 1, to a previously established and clinically accepted model (Fig. 2), referred to as the reference model. This reference model is based on one lumped outflow resistance $R_{{\text {out}}}$ and a pressure-dependent compliance. Several full model modifications will be described and considered.

### Characteristics of infusion resistances and compliance

Infusion tests in humans provide crucial insights and basic characteristics for models of CSF dynamics, including validated pressure ranges. A linear relationship between steady state pressure elevation and infusion rate, i.e. the assumption of a pressure-independent $R_{\mathrm{out}}$, has been shown to be valid from baseline ICP ($p_{\mathrm{base}}$) up to $p_{\mathrm{base}} +12 \, \text {mmHg}$ [34]. At higher pressure increases from baseline ($> 15 \, \text {mmHg}$ increase), this assumption does not hold. Further, the craniospinal compliance is typically assumed to be inversely dependent on the ICP. This assumption is for most subjects valid from a threshold ICP ($p_{\mathrm{thres}}$) of approximately 11 mmHg and higher. For lower ICPs, compliance should be modelled as constant [35, 36]. These insights are reflected by the model proposed here. We assume the inverse compliance model for ICP above baseline pressure and a constant compliance for lower ICP, while the pressure independent property of $R_{{\text {out}}}$ sets the upper ICP validity limit to approximately 23–26 mmHg.

### Mathematical model of CSF pressure dynamics under infusion

We modeled the CSF pressure in the SAS ($p_0$) by an ordinary differential equation (ODE), and the (arteriolar) PVS ($p_1$) described by an algebraic expression. For a schematic overview of the model compartments and routes, see Fig. 1. Our model extends on previous models [27, 28] of CSF pressure, flow and compliance within the intracranial compartment, by also computing PVS pressure and by including additional outflow pathways. To account for flow into the PVS from both capillaries and the SAS, the PVS is modeled as a pressure compartment similar to the SAS. This compartment represents the PVS from the arteriole to precapillary segments at the end of the paraarterial tree as modeled by Faghih and Sharp [11].

Assuming a lumped compliance *C* of the system, the ODE describing the PVS pressure can be reduced to an algebraic expression. The problem then reads: find $p_0 = p_0(t)$ and $p_1 = p_1(t)$ for $t \ge 0$ such that

1 $$\begin{aligned} C(p_0)\frac{\partial p_0}{\partial t}&= Q_{\mathrm{in}} + \frac{1}{R_{\mathrm{AG}}}(p_{\mathrm{d}} - p_0) + \frac{1}{R_{\mathrm{crib}}}(p_{\mathrm{crib}} - p_0) + \frac{1}{R_{\text {aPVS}}}(p_1 - p_0) \nonumber \\&\quad + \frac{1}{R_V}(p_1 - p_0), \end{aligned}$$

2 $$\begin{aligned} 0&= \frac{1}{R_{\text {cap}}}(p_{\mathrm{cap}} - p_1) + \frac{1}{R_{\text {V}}}(p_0 - p_1) + \frac{1}{R_{\text {aPVS}}}(p_0 - p_1). \end{aligned}$$

The (given) constant counter pressures for the AG, cribriform plate, and capillaries are denoted by $p_{\mathrm{d}}, p_{\mathrm{crib}}$ and $p_{\mathrm{cap}}$, respectively. The corresponding resistances are denoted by $R_{\mathrm{AG}}, R_{\mathrm{crib}}$ and $R_{\mathrm{cap}}$. $R_{\mathrm{aPVS}}$ is the resistance to flow between the SAS ($p_0$) and PVS ($p_1$) compartments through paravascular spaces. $\frac{1}{R_{\text {cap}}}(p_{\mathrm{cap}} - p_1) = Q_{cap}$ is fluid secretion across the blood–brain barrier. Due to uncertainty of the magnitude of this term, a variation in the model using a constant filtration of water from the capillaries will also be considered, as transport across the blood–brain barrier is a highly regulated and complex process [37]. $Q_{\mathrm{in}}$ is the sum of CSF production and fluid infusion. Possible capillary filtration is not included in $Q_{\text {in}}$, but rather as a separate term in Eq. (2). The total resistance $R_{\mathrm{V}}$ to flow going from arterial PVS out of the brain along venous PVS is given by:

3 $$\begin{aligned} R_{\mathrm{V}} = \left( \frac{1}{R_{\mathrm{gaps}}} + \frac{1}{R_{\mathrm{IEG,a}} + R_{\mathrm{ECS}}+ R_{\mathrm{IEG,v}}} \right) ^{-1} + R_{\mathrm{vPVS}}. \end{aligned}$$

Furthermore, the total resistance as seen from the SAS through the paravascular pathway (glymphatic pathway) can be computed as

4 $$\begin{aligned} R_{\mathrm{glymph}} = R_{\mathrm{aPVS}} + \left( \frac{1}{R_{\mathrm{cap}}} + \frac{1}{R_{\mathrm{V}}} \right) ^{-1}. \end{aligned}$$

### Model parameters

This section describes the model parameters including estimation of resistances between the different pressure compartments. These parameters are summarized in Table 1.

**Table 1:** Model 1 default parameters (corresponding to full model) cf. Fig. 1 and Eqs. (1), (2)

Parameter	Symbol	Value	Unit	References
Dural sinus pressure	$p_{{\text {d}}}$	8.4	mmHg	Eq. (17 ) [61–63]
Cervical lymph pressure	$p_{{\text {crib}}}$	0	mmHg	[64]
Pial sleeves pressure	$p_{{\text {V}}}$	8.4	mmHg	Model assumption
Hydrostatic capillary pressure	$p_{cap,h}$	35	mmHg	[73]
Effective capillary pressure	$p_{\mathrm{cap}}$	20	mmHg	Eq. (19)
Reference pressure	$p_{{\text {r}}}$	9	mmHg	[39]
Threshold pressure	$p_{{\text {thres}}}$	11	mmHg	[34]
AGs resistance	$R_{{\text {AG}}}$	10.81	mmHg/(mL/min)	[57]
Paraarterial resistance	$R_{{\text {aPVS}}}$	1.14	mmHg/(mL/min)	[11]
Arterial IEG resistance	$R_{\mathrm{IEG,a}}$	0.57	mmHg/(mL/min)	Eq. (15) [50]
Venous IEG resistance	$R_{\mathrm{IEG,v}}$	0.64	mmHg/(mL/min)	Eq. (15) [50]
ECS resistance	$R_{\mathrm{ECS}}$	0.57	mmHg/(mL/min)	Eqs. (12, 13) [14, 49]
Capillary gaps resistance	$R_{{\text {gaps}}}$	32.24	mmHg/(mL/min)	Eq. (8) [45]
Paravenous resistance	$R_{{\text {v}}}$	$1.75 \times 10^{-3}$	mmHg/(mL/min)	[11]
Lumped model resistance	$R_{{\text {V}}}$	1.69	mmHg/(mL/min)	Eq. (3)
Cribriform plate resistance	$R_{{\text {crib}}}$	67	mmHg/(mL/min)	Eq. (3)
Capillary wall resistance	$R_{{\text {cap}}}$	125.31	mmHg/(mL/min)	Eq. (16)
Subarachnoid space elastance	*E*	0.2	${\text {mL}}^{-1}$	[27]
CSF production rate	$Q_{{\text {prod}}}$	0.33	mL/min	[40–43]
Infusion rate	$Q_{{\text {inf}}}$	1.5	mL/min	[27]

#### Compliance

We assume that the craniospinal compliance is inversely proportional to the difference between the SAS pressure ($p_0$) and a reference pressure ($p_r$) for SAS pressures above a certain threshold $p_{\mathrm{thres}}$ but constant below said threshold pressure [25, 38]:

5 $$\begin{aligned} C(p_0) = {\left\{ \begin{array}{ll} \frac{1}{E\cdot (p_0 - p_r)} &{} {\text {if}} \quad p_0 \ge p_{\mathrm{thres}}\\ \frac{1}{E\cdot (p_{\mathrm{thres}} - p_r)}&{} {\text {if}} \quad p_0 < p_{\mathrm{thres}}. \end{array}\right. } \end{aligned}$$

*E* is known as the elastance coefficient. We set $E = 0.2 \, {\text {mL}}^{-1}$ in accordance with reported values [27]. While the physical interpretation of the reference pressure is less immediate [27], we used the value $p_r = 9 \, \text {mmHg}$ as reported by Jacobsson et al. [39]. Furthermore, for $p_0 < p_{\text {thres}}$, where $p_{\text {thres}}\approx 11 \, \text {mmHg}$, the assumption of a linear relationship between pressure and volume changes [39], implies a constant *C* at low pressures. At $p_0 < p_{\text {thres}}$, we thus set the compliance to the baseline compliance in the system.

#### CSF production

In medical textbooks, CSF production rate is normally reported at roughly 500 mL/day [40], which corresponds to 0.35 mL/min. Some studies report a production of around 0.4 mL/min [41, 42], while production rates as low as 0.19 mL/min have been observed in healthy elderly [43]. In this work, we used a production rate of $Q_{\text {prod}} = 0.33 \, \text {mL/min}$.

#### Resistance to flow in the PVS

We assumed the paravascular tree resistance model proposed by Faghih and Sharp [11] to represent the paraarterial space (including precapillaries) and the paravenous space. The resistance in these spaces were reported to be $R_{{\text {aPVS}}} = 1.14 \, \text {mmHg/(mL/min)}$ and $R_{{\text {vPVS}}} = 1.75 \times 10^{-3} \, \text {mmHg/(mL/min)}$, respectively.

#### Resistance to flow along the microcirculation ($R_{\text {gaps}}$)

Resistance to flow is given by the ratio of pressure drop ($\Delta p$) to the flow rate in the given geometry

6 $$\begin{aligned} R = \frac{\Delta p}{Q}. \end{aligned}$$

In this work, we will assume the PVS to form a circular annulus around the vessel wall. The flow rate in an annular section is given by [44]:

7 $$\begin{aligned} Q = \frac{\Delta p}{L}\frac{\pi }{8 \mu } \left[ r_2^4 - r_1^4 - \frac{(r_2^2 - r_1^2)^2}{{\text {log}}(r_2/r_1)} \right] , \end{aligned}$$

where $\mu$ is fluid viscosity, $r_1$ and $r_2$ are inner and outer radius of the annulus, respectively, and $\Delta p$ is the pressure drop over the section of length *L*. By combining Eqs. (6) and (7), the flow resistance in each PVS can be computed as

8 $$\begin{aligned} R = \frac{8 \mu }{\pi r_1^4} \left[ \frac{L}{(k^{-4} - 1) - \frac{(k^{-2}-1)^2}{{\text {log}}(k^{-1})}} \right] , \end{aligned}$$

where $k=\frac{r_1}{r_2}$ is the ratio between inner and outer radius.

The paravascular resistance model proposed by Faghih and Sharp [11] ended at the precapillary level, where precapillary diameters were $12.2{-}12.5 \, \upmu \text {m}$. In a 1D network of the microvasculature, Payne and El-Bouri [45] reported blood vessel radius and length based on the modeling framework by Boas et al. [46]. The 1D network started at the arteriole level with a vessel radius of $12 \, \upmu \text {m}$ and branched out to 64 capillaries each with a radius of $4 \, \upmu \text {m}$. At the final venule, the radius was $15 \, \upmu \text {m}$. We assumed this tree to form the microcirculation and added small gaps of 100 nm [11] around the vessels of varying size allowing for CSF flow along the microvasculature. The gap size of 100 nm, as used by Faghih and Sharp [11], is based on experimental work by Bedussi et al [31] who found paravascular spaces in the microcirculation to be located just outside the endothelial layer, which coincide with the basement membrane of the capillaries with a thickness of 50–100 nm [47].

The 1D network model [45] consists of a single tree branching from the first generation of arteriole (precapillaries), splitting at each generation until reaching the middle capillary level (generation 7). At this level, the tree consists of 64 capillaries only. After the capillary branch, the tree joins back together to the last venule (postcapillary), such that the total tree consists of 13 generations. The total number of capillaries in the brain is estimated to be around 100 billion [48]. We therefore assumed the brain to have $N = 1.5625$ billion trees, with 64 capillaries in each tree, each identical to the one used by Payne and El-Bouri [45].

The branching tree can be viewed as a combination of parallel and series circuits. To compute the total flow resistance $R_{\text {gaps}}$ in the gaps around the microcirculation, we computed the resistance of each generation and added these together to form the cumulative resistance for a single tree, $R_i$:

9 $$\begin{aligned} R_i = R_{A1} + \frac{R_{A2}}{2} + \dots + \frac{R_C}{64} + \frac{R_{V6}}{32} + \dots + R_{V1}, \end{aligned}$$

where $R_{Ai}$ denotes the resistance of the i’th paraarteriolar generation, $R_{Vi}$ denotes the resistance of the i’th paravenular generation, and $R_C$ denotes the resistance of the paracapillary gaps. Assuming a parallel configuration of the networks, the total resistance of the gaps around the microcirculation is given by dividing the resistance of a single tree by the number of trees *N*:

10 $$\begin{aligned} R_{\text {gaps}} = R_i/N . \end{aligned}$$

#### Resistance to flow in the ECS ($R_{\text {ECS}}$)

Estimating the resistance posed by the ECS is a challenge as the ECS is a porous medium in 3D permeated by an almost space-filling network of vessels. Although the permeability in the ECS has already been measured [14], the average length and the combined area for flow between arteries/arterioles and veins/venules needs to be found to compute resistance. To compute the resistance of the ECS, we therefore computed ISF flow according to Darcys law in a realistic 2D domain as described in the following.

**Fig. 3:**
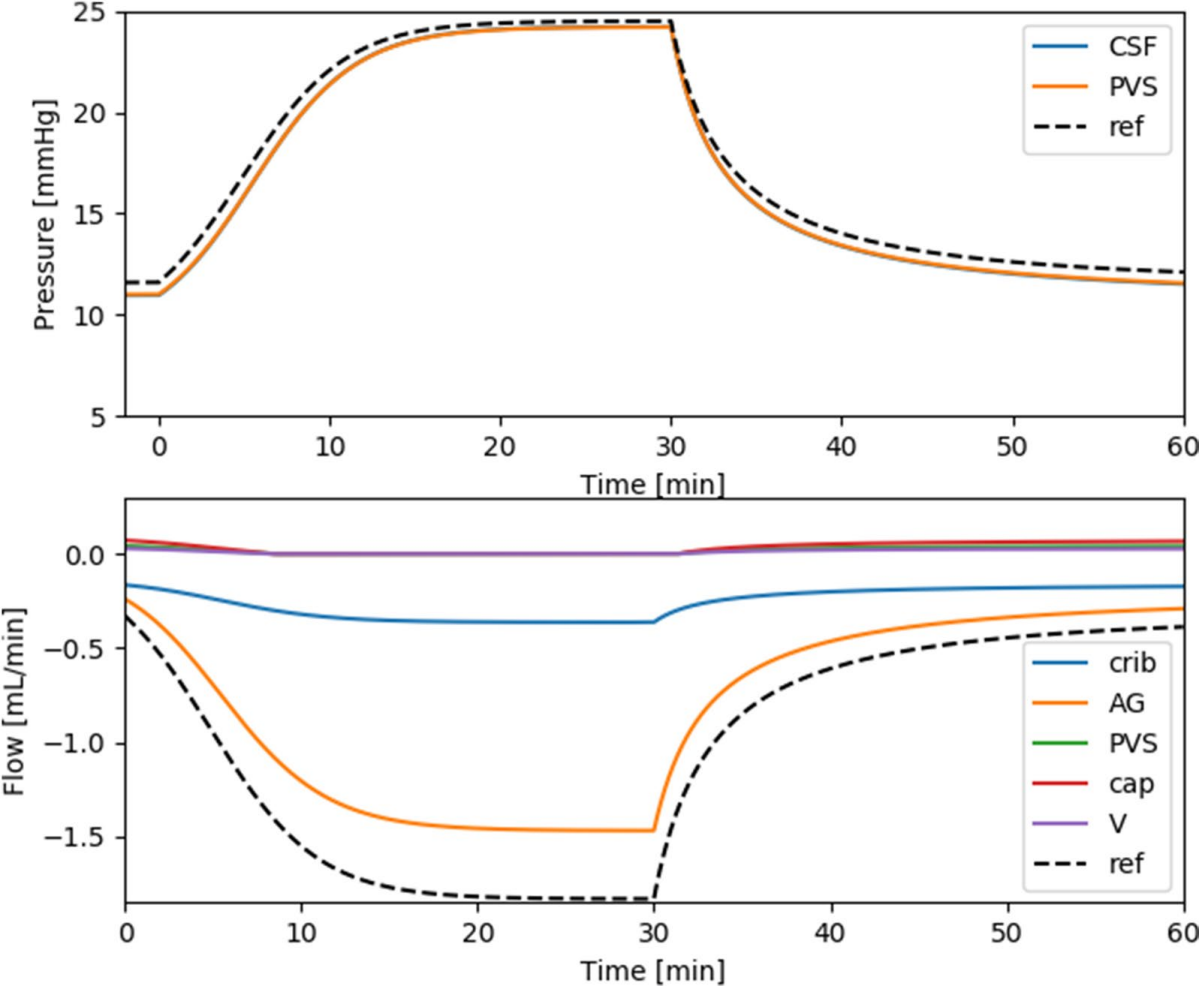
CSF pressure and outflow during a standard infusion test. The arachnoid granulations dominated outflow both under baseline and (elevated) plateau ICP, with 60.0% and 80.3% of the fluid leaving through the granulations. The cribriform plate plays a much less prominent role at plateau than at baseline ICP (40.0% vs. 19.7%). Secretion from capillaries and flow in PVS are small compared to flow to the AG and cribriform plate. At baseline, (arterial) paravascular flow is small at 0.04 mL/min into the SAS. The capillary secretion rate is 0.07 mL/min. Flow in paravenous spaces will thus be 0.03 mL/min to balance out the capillary secretion (data now shown). During infusion, capillary filtration ceases due to the assumption that net absorption is impossible

Adams et al. [49] reported the mean distance between arterioles and venules to be $280 \, \upmu \text {m}$. The same authors also show several layers of the cortex with distributions of arterioles and venules. Based on previous estimations [49, 50], we assumed arteriole diameters of $30 \, \upmu \text {m}$ and venule diameters of $40 \, \upmu \text {m}$, and created a computational grid of the 2D slice as shown in Figure 3d by Adams et al. [49]. The domain had dimensions $L = 3.91 \, \text {mm}$ and $H = 2.81 \, \text {mm}$ and included 125 arteries and 50 venules represented as small holes in the domain. The domain is shown in Additional file 1. In this computational domain, we solved for ECS flow according to Darcy’s law [51] driven by a pressure gradient between arteriole and venule PVS:

11 $$\begin{aligned} q = -\frac{\kappa }{\mu }\nabla p \end{aligned}$$

where *q* is Darcy velocity, $\mu$ is the dynamic viscosity of the fluid, and $\nabla p$ is the pressure gradient driving flow. On outer boundaries we imposed symmetry (Neumann) conditions. The resulting flow rate between arterioles and venules is given by

12 $$\begin{aligned} Q = \int _{\partial \Omega _a} -q \cdot n \, \mathrm {d}s, \end{aligned}$$

where ${\partial \Omega _a}$ the total arteriole surface, and *n* is the outward normal (i.e. pointing into the arteriole). *Q* is thus all flow leaving the arterioles, and will be equal to the total flow entering venules. It should be noted that *Q* is the total flow per unit depth in the 2D-slice we used as computational domain, and has units ${\text {m}}^2/\text {s}$. The depth *D* of the 2D-domain was assumed such that the volume of the cube was equal to a brain volume of $1 \, {\text {dm}}^3$, i.e. $D \times L \times H = V_{\text {brain}}$. Rearranging, and solving for the depth gave $D = 90.67 \, \text {m}$. Independent of the size of the pressure gradient in the ECS, Eqs. (6) and (12) can be combined to find the resistance by

13 $$\begin{aligned} R = \frac{\Delta p}{Q} \end{aligned}$$

which was used to calculate the total resistance of the ECS. The computed resistance is thus independent of the pressure gradient used to compute flow in Eq. (12).

#### Inter-endfeet gaps

To calculate the IEG resistance, we took advantage of the same geometry as in the previous section. To enter the ECS, fluid has to go either through inter-endfeet gaps (IEG), or through AQP-4 channels via the intracellular space. Asgari et al. [50] showed that water transport through IEG was the most likely of the two, and we therefore assumed this route for water transport directly to the ECS. The height of the IEG is $h = 20-30 \, \text {nm}$ and the thickness of the endfoot is $T = 1 \, \upmu \text {m}$ [50]. The height of the IEG was set to 24 nm on arterioles and 31 nm on venules [52]. To calculate a total resistance for these clefts, the number of clefts or alternatively the total area they make up needs to be known. In the spatial model by Jin et al. [52], 2 IEG on a domain representing 1/6 of a vessel was considered, suggesting 12 IEG on the horizontal cross section of a vessel. For each cross section of capillaries, Mathiisen et al. [53], found 2.5 such IEG on average making up to a total cleft area of 0.3 % of the vessel area. Therefore, for the arterioles and venules in the geometry we used in the previous subsection, we calculated the expected number of IEG on a cross section of a vessel, $n_i$, by solving $h_i \, n_i = 0.003 \times 2\pi r_i$, where $r_i$ is the vessel radius. Here, the subscript *i* denote type of vessel, i.e. arteriole or venule.

In previous modelling work, the length of the IEG was set to $l = 5 \, \upmu \text {m}$ [54]. However, when assuming $n_i$ IEG on average along the cross section of a vessel, whether they form a few long continuous channels or several discontinuous channels of $5 \, \upmu \text {m}$ along the vasculature is equivalent. The resistance through one IEG is given by $\frac{12 \mu T}{h^3 \,l}$ [50], and the total parallel outflow resistance through IEG of one vessel is given by

14 $$\begin{aligned} R_{\text {IEG}} = \left( \frac{1}{R_{\text {cleft,i}}} n_i\right) ^{-1} = \frac{12 \mu T}{h_i^3 l \,n_i}, \end{aligned}$$

where $N_c$ is the total number of clefts on all vessels. Due to the assumption of a total cleft area $A_{\text {IEG}} \, l \,n_i$ of 0.3 % of the vessel area, and a constant height, the product $n_i\, l$ needs to be constant regardless of changes in one of the two. Therefore we assumed each vessel to have $n_i$ continuous clefts along the vasculature, and set $l = D$, where *D* is the depth of the 2D domain as used in the previous subsection. The domain consisted of $N_a = 125$ arterioles, and $N_v = 50$ venules, and by assuming Eq. (14) the IEG flow resistance on all vessels combined can be computed as

15 $$\begin{aligned} R_{\text {IEG,i}} = \left( \frac{1}{R_{\text {cleft,i}}} n_i\right) ^{-1} = \frac{12 \mu T}{h^3 l \,n_i N_i}, \end{aligned}$$

for $i = a, v$ (arteriole and venule). The surface area of the arteries/arterioles and veins/venules (the cerebral vasculature excluding capillaries) was computed to be $1.64 \, {\text {m}}^2$ in the geometry we used.

The parameters used to calculate ECS and IEG resistance are summarized in Additional file 2: Table S1.

#### Resistance and pressure for outflow into dural sinus or lymphatics

In AG tissue, the hydraulic conductivity, $L_p$ has been estimated experimentally at $4.52 \, \upmu \text {L/min} \, {\text {per mmHg/cm}}^2$ ($5.66 \times 10^{-9} \, \text {m/(Pa s)}$) [55], and later adjusted to $92.49 \, \upmu \text {L/min}$ per ${\text {mmHg/cm}}^2$ ($1.16 \times 10^{-7} \, \text {m/(Pa s)}$) with serum free media [56]. The resistance *R* relates to the hydraulic conductivity $L_p$ by

16 $$\begin{aligned} R = \frac{1}{L_p A}, \end{aligned}$$

where *A* is the surface area. A cranial AG surface area of $1 \, {\text {cm}}^2$ [57] yields $R_{AG} = 221.24 \, \text {mmHg/(mL/min)}$ without and $R_{AG} = 10.81 \, \text {mmHg/(mL/min)}$ with serum free media. The value 10.81 mmHg/(mL/min) seems the more reasonable as median total resistance in healthy elderly has been reported to be 8.6 mmHg/mL/min [3]. We assume spinal absorption to be included in $R_{AG}$. Spinal absorption could account for as much as 15–35% of total absorption [58–60].

The AG counter pressure is represented by the dural sinus pressure $p_{\text {d}}$. Measurements of $p_{\text {d}}$ in healthy controls are scarce, instead we relate $p_{\text {d}}$ to the central venous pressure (CVP) as follows:

17 $$\begin{aligned} p_d = CVP + \Delta p_{CVP-p_d}, \end{aligned}$$

where $\Delta p_{CVP-p_d}$ represents the pressure difference between CVP and dural sinus pressure due to venous flow and flow resistance. Holmlund et al [61] reported CVP in healthy controls in supine position to be 4.2 mmHg. Avasthey [62] measured venous pressure at various sites in the venous system and found a pressure increase of approximately 2.2 mmHg from the CVP (measured in the right atrium) to the internal jugular vein at the level of the ear. Further, Bateman and Bateman [63] reported the pressure increases in the sagittal and straight sinus of controls to be 1.3 and 0.7 mmHg, respectively. Adding the pressure increases from the right atrium to the sagittal sinus we can approximate $\Delta p_{CVP-p_d} = 4.2 \, \text {mmHg}$ and $p_{\text {d}} = 8.4 \, \text {mmHg}$. It should be noted that both ICP and the dural venous pressure changes with body position [61], and that we only considered the supine position.

#### Resistance and pressure for outflow via the cribriform plate

The CSF from the SAS may be transported through the cribriform plate into extracranial lymphatic vessels [64]. The total outflow resistance has been calculated via infusion tests with and without blockage of the cribriform plate in sheep [20]. Assuming an estimated outflow resistance $R_{{\mathrm{out}}_{\mathrm{pre}}}$ before blockage and $R_{{\mathrm{out}}_{\mathrm{post}}}$ after blockage, the cribriform plate resistance $R_{\text {crib}}$ can be estimated by assuming two parallel outflow routes ($R_{{\mathrm{out}}_{\mathrm{post}}}$ and $R_{\text {crib}}$):

18 $$\begin{aligned} \frac{1}{R_{{\text {crib}}}} = \frac{1}{R_{{\text {out}}_{{\text {pre}}}}} - \frac{1}{R_{{\text {out}}_{{\text {post}}}}} . \end{aligned}$$

Equation (18) yields a resistance of 67 mmHg/(mL/min). We assume that CSF leaves the olfactory bulb via the cribriform plate and enters the nasal epithelium where it is absorbed by the peripheral lymphatic system [20, 65]. Further, we assume that the outflow across the cribriform plate is driven by the difference between ICP and the tissue pressure within the nasal epithelium. The pressure in the nasal epithelium is to our knowledge unknown. However, the nasal epithelium is in direct contact with the nasal cavity where the pressure is close to atmospheric. (The pressure gradient in the nasal cavity is routinely measured by rhinomanometry and the pressure difference between the nostrils and nasopharynx is $>-\,2 \, \text {mmHg}$ during inspiration and $<2 \, \text {mmHg}$ during expiration [66].) In our model, $p_{\text {crib}}$ was set to 0 mmHg.

#### Resistance and pressure related to filtration/absorption across capillaries

The total hydraulic (or vascular) conductivity $L_p$ of the blood vessel walls throughout the brain has been reported to be approximately $10^{-13}$ (m/Pa s) [67–69]. Assuming a surface-to-volume ratio (S/V) of $10{,}000 \, {\text {m}}^2/{\text {m}}^3$ [67, 70] and that the brain has a volume of about $1 \, {\text {dm}}^3$ yields a total vascular surface area of $\text {A} = 10 \, {\text {m}}^2$. Combining these values with (16), the flow resistance over the blood vessel walls, $R_{\text {cap}}$, can be estimated as 125.31 mmHg/(mL/min) ($10^{12} \, {\text {Pa s/m}}^3$).

In peripheral tissue fluid flow across the capillary wall is described by Starling’s law. Fluid is filtrated out on the arterial side of the capillary bed and reabsorbed on the venous side. The difference between filtrated and absorbed fluid is the net flow. Starling’s law has been applied in mathematical models of fluid exchange in the brain [67, 71, 72]. The blood–brain barrier ensures low ion permeability over the capillary wall in the brain [21, 23]. However, fluid exchange across this barrier is still a combination of hydrostatic and osmotic forces [21, 23] which are taken into account to estimate the effective capillary pressure ($p_{\mathrm{cap}}$) used in our model.

19 $$\begin{aligned} p_{\mathrm{cap}} = {\left\{ \begin{array}{ll} p_{\mathrm{cap,h}} - \sigma (\pi _{cap} - \pi _{PVS}), &{} \qquad p_{\mathrm{cap}} \ge p_{1}, \\ p_{1} &{} \qquad p_{\mathrm{cap}} < p_{1}, \end{array}\right. } \end{aligned}$$

where the hydrostatic capillary pressure $p_{cap,h} = 35 \, \text {mmHg}$ [73], the reflection coefficient $\sigma = 1$ [74], the oncotic blood pressure $\pi _{cap} = 25 \, \text {mmHg}$ [74], and the oncotic tissue pressure $\pi _{PVS}$ is set to 40% of $\pi _{cap}$ (10 mmHg) [74], resulting in $p_{\mathrm{cap}} = 20 \, \text {mmHg}$. Note that the contribution from the electrolytes to the osmotic pressure, both on the vascular and interstitial side, is not included and thus assumed to be equal and constant. Finally, for the purpose of our simulations we assume that these relationships hold for pressures up to $p_{\mathrm{cap}} = p_{1}$, i.e., we do not allow net reabsorption into capillaries from PVS. We acknowledge that this model does not include an increase in venous capillary pressure with increasing ICP.

### Model variations

To investigate the sensitivity of the predicted intracranial pressures and associated flow between compartments with respect to different parameter regimes, we consider a set of model variations. Each variation is labelled and described below, and summarized in Table 2.

**Table 2:** Overview of parameters and modifications for models 0–9 used in the study

Model	$R_{\text {AG}}$ [$\frac{{\text {mmHg}}}{{\text {mL/min}}}$]	$R_{\text {crib}}$ [$\frac{{\text {mmHg}}}{{\text {mL/min}}}$]	$R_{\text {aPVS}}$ [$\frac{{\text {mmHg}}}{{\text {mL/min}}}$]	$R_{\text {cap}}$ [$\frac{{\text {mmHg}}}{{\text {mL/min}}}$]	$R_{\text {V}}$ [$\frac{{\text {mmHg}}}{{\text {mL/min}}}$]	Additional notes
0	8.6	$\infty$	$\infty$	$\infty$	$\infty$	–
1	10.81	67.0	1.14	125.31	1.69	–
2	10.81	67.0	1.14	125.31	1.69	Pial sleeves outflow
3	10.81	67.0	1.14	n/a	1.69	$Q_{\text {cap}} = \text {const.}$
4	10.81	67.0	$\infty$	$\infty$	$\infty$	–
5	$\infty$	67.0	1.14	125.31	1.69	–
6	21.62	67.0	1.14	125.31	1.69	–
7	10.81	67.0	1.14	125.31	2.65 × 10^−3^	$R_{\text {gaps}} = 1.43 \times 10^{-3}$

All other parameters, cf. Table 1, were kept constant. Model 7 also included a direct route to cervical lymphatics to distinguish it from the full model (model 1)

#### Reference model (model 0)

As a reference model, we used the median value of $R_{\text {out}} = 8.6 \, \text {mmHg/(mL/min)}$ as reported by Malm et al. [3] in a cohort of healthy subjects. In simulations this was accomplished by assuming AG to be the only outflow route, thus setting $R_{\text {AG}} = 8.6 \, \text {mmHg/(mL/min)}$. All other resistances *R* were set to $\infty$, i.e., $1/R = 0$.

#### Full model (model 1)

The model described in Fig. 1 and by Eqs. 1 and 2 is referred to as the full model, or model 1. In this model, all pathways considered were assumed possible. The models (2–7) below are described as modifications to the full model.

#### Direct glymphatic route to cervical lymphatics (model 2)

In this model we assumed an exit pathway from paravenous spaces directly to cervical lymphatics rather than a return to the SAS. This could be e.g. pial sleeves [32], leading directly to cervical lymph as envisioned in early works regarding the glymphatic theory [5, 29]. The outflow pressure after the paravenous spaces (pial sleeves pressure) was assumed comparable to the AG counter pressure at $p_V = 8.4 \, \text {mmHg}$ because both absorption sites are located in the same anatomical region assumed to be separated from the CSF compartment. We assume that there are high resistive ducts after intracranial venous PVS generating the additional pressure drop from the ICP and PVS pressure. The model was tested by changing the second term in Eq. (2) to $\frac{1}{R_V}(p_V - p_1)$.

#### Constant capillary filtration (model 3)

In this model, we assumed a small net capillary filtration independent of ICP. Even though capillaries constantly filtrate water [21], the net flow rate over the capillary wall, if any [75, 76], is hard to quantify. A constant capillary flow could be justified by the fact that active transportation is independent of both hydrostatic and osmotic forces [76] and, furthermore, large osmotic forces always dominate and adjust to small changes in hydrostatic forces [23]. Hladky & Barrand [23] suggest that 685 mL/day would be a high estimate of net fluid production by the capillaries. In this model, we set a net capillary filtration close to half of that at 0.16 mL/min, but also ensured that we would reach the same conclusions with values in the range 0–0.3 mL/min.

#### Glymphatic pathway eliminated (model 4)

The glymphatic circulation and its role in the net clearance of CSF out of the intracranial compartment is disputed [77]. We tested the effect of eliminating glymphatic function in our model by letting all flow exit through either the cribriform plate or the AG.

#### AG pathway eliminated (model 5)

This model represents a complete dysfunction in the AG, effectively eliminating the AG as a pathway. We tested whether other outflow routes could compensate, and to what extent ICP would be increased.

#### Increased AG resistance (model 6)

Experimentally, the AG outflow resistance has been found to vary by a factor 20, depending on the fluid properties. We therefore also tested an increased resistance of 21.62 mmHg (corresponding to twice the value of the full model) to test the model sensitivity with respect to this parameter. This variation also tested whether other outflow routes could compensate for an increase in $R_{\text {AG}}$, in contrast to a complete elimination as in model 5.

#### Extended capillary gaps (model 7)

Mestre et al. [8] demonstrate PVS collapse after fixation. This observation entails that a gap size of 100 nm at the precapillary level, as used by Faghih and Sharp [11] and measured after fixation, may be an underestimation. If we rather assume a linear change in area ratio between cross sectional PVS and lumen (going from 1.26 to 0.13 [78] over 13 generations), resistance is vastly reduced. For the different vessel radii reported by Payne and El-Bouri [45], we set $r_{\text {PVS}} = \sqrt{A_{\text {PVS}} + r_1^2}$, and computed the resistance in Eq. (8) with $k=\frac{r_1}{r_{\text {PVS}}}$.

### Computation of steady state values

In steady state, the left hand side of Eq. (1) is zero and independent of compliance. We calculated the steady state solution of the CSF pressure in the SAS as a function of the infusion rate $Q_{\text {inf}}$. The function takes the form

20 $$\begin{aligned} p_0(Q_{\text {inf}}) = R_0Q_{\text {inf}} + p_{0,{\text {base}}}, \end{aligned}$$

In a clinical infusion test, $R_{\text {out}}$ is measured in the SAS, and thus corresponds to our estimation of $R_0$. $p_{0,{\text {base}}}$ is the ICP in the SAS at baseline ICP. For each model we report both $R_0$ and $p_{0,{\text {base}}}$ such that ICP can be computed for any arbitrary infusion rate. In addition, we calculate the pressures and corresponding flows at rest and at peak ICP resulting from an infusion test with $Q_{\text {inf}} = 1.5 \, \text {mL/min}$.

### PVS pressure and velocity estimation

Following [11], we modeled the paraarterial tree as stemming from three branches of the arterial tree. The paraarterial model started at generations 18, 16 and 16 after the middle cerebral artery, anterior cerebral artery, and second posterior cerebral artery with diameters $d_0 = 100.16$, $d_1 = 97.42$ and $d_2 = 97.42 \, \upmu \text {m}$ (and radii $r_0$, $r_1$ and $r_2$), respectively [11]. Following the assumption that arterial PVS area is $c = 1.26$ times that of the lumen area [78], the total PVS cross-sectional area at the starting branch is:

21 $$\begin{aligned} A_{\text {PVS}} = c(2^{18} \pi r_0^2 + 2^{16}\pi r_1^2 + 2^{16} \pi r_2^2) = 38 {\text { cm}}^2. \end{aligned}$$

We can thus calculate the average velocity at the base of the arterial PVS by

22 $$\begin{aligned} v_{\text {pvs}} = Q_{\text {PVS}}/A_{\text {PVS}} = \frac{1}{R_{\text {aPVS}}A_{\text {PVS}}}(p_0 - p_1). \end{aligned}$$

for ICP $p_0$ and PVS pressure $p_1$, and negative values indicate flow from PVS into the SAS. In our Table 3, we do not explicitly report PVS pressure, but for any model the pressure can be deduced from the tables by

23 $$\begin{aligned} p_1 = p_0 - RQ, \end{aligned}$$

where Q is the flow rate from SAS to PVS (negative for flow from PVS to SAS), and R is the resistance between the compartments. Similar relations can be used to explicitly compute pressures in all other compartments as well.

**Table 3:** Effect of modifications to steady state results of the model on ICP and flow

Mod	ICP ($p_0$) [mmHg]	AG flow [mL/min]	PVS flow [mL/min]	Crib flow [mL/min]	Cap flow [mL/min]	$R_0$ [$\frac{{\text {mmHg}}}{{\text {mL/min}}}$]
0	11.66 (24.16)	− 0.33 (− 1.83)	n/a	n/a	n/a	8.60
1	10.99 (24.28)	− 0.24 (− 1.47)	0.04 (0.00)	− 0.16 (− 0.36)	0.07 (0.00)	8.67
2	8.98 (12.21)	− 0.05 (− 0.35)	− 0.14 (− 1.30)	− 0.13 (− 0.18)	0.09 (0.07)	2.16
3	11.81 (25.77)	− 0.31 (− 1.61)	0.10 (0.10)	− 0.18 (− 0.38)	0.16 (0.16)	9.31
4	10.32 (24.28)	− 0.18 (− 1.47)	n/a	− 0.15 (− 0.36)	n/a	9.31
5	22.11 (122.61)	n/a	0.00 (0.00)	− 0.33 (− 1.83)	0.00 (0.00)	67.00
6	12.71 (36.28)	− 0.20 (− 1.29)	0.03 (0.00)	− 0.19 (− 0.54)	0.06 (0.00)	14.47
7	8.42 (8.43)	0.00 (0.00)	− 0.20 (− 1.70)	− 0.13 (− 0.13)	0.09 (0.09)	0.00

Results are reported as max/min where max is the value at plateau ICP (shown within parenthesis), while min is the value at baseline ICP. Negative values indicate flow out from the SAS and into the given compartment. For capillary flow, positive values indicate secretion of fluid from the capillaries

### Numerical methods and setup

The system (1)–(2) of ODEs was solved in Python using the Scipy [79] (version 1.1.0) ODE solver, odeint. At each time step, the solver recognizes characteristics of the linear system and is adaptive with respect to solver method (Adams or BDF), order, and time step. As initial conditions, we set $p_0(0) = p_1(0) = 8 \, \text {mmHg}$, and we assumed a CSF production $Q_{\text {prod}} = 0.33 \, \text {mL/min}$ (see 1). The equations were first solved to reach a steady state (t = 60 min) before adding the infusion fluid $Q_{\text {inf}} = 1.5 \, \text {mL/min}$ for 30 min. Thus in Eq. (1), we set

24 $$\begin{aligned} Q_{\text {in}} = {\left\{ \begin{array}{ll} Q_{\text {inf}} + Q_{\text {prod}}, &{} {\text {for }} 60 {\text { min}} \le t \le 90 {\text { min}}, \\ Q_{\text {prod}}, &{} {\text { otherwise}}. \end{array}\right. } \end{aligned}$$

We note that the given $Q_{\text {prod}}$ does not include the possible capillary filtration included in Eq. (2).

## Results

In the three following subsections, results for the full model (model 1) are presented along with comparison to the reference model. In the fourth subsection results from modifications to the full model (models 2–7) are presented.

### Flow resistances

#### Capillary gaps

For the single 1D network [45], the resistance was computed to be

25 $$\begin{aligned} R_i & = R_{A1} + \frac{R_{A2}}{2} + ... + \frac{R_C}{64} + \frac{R_{V6}}{32} \\ & \quad + ... + R_{V1} = 5.04 \times 10^{10} \frac{{\text {mmHg}}}{{\text {(mL/min)}}}. \end{aligned}$$

Assuming the parallel configuration of the networks, the total resistance of the annular gaps around the microcirculation is found by dividing by the number of trees:

26 $$\begin{aligned} R_{\text {gaps}} = R_i/N = 32.24 {\text { mmHg/(mL/min)}}. \end{aligned}$$

This resistance is far too high to sustain capillary gap flow through the glymphatic route and returning to the SAS, as any considerable flow would require local pressure differences of several mmHg in the SAS. For instance, a flow of 0.13 mL/min, representative of interstitial fluid perfusion in humans [11], would require a pressure drop of 4.19 mmHg along the capillary gaps. The maximal estimated transmantle pressure gradient of 0.03 mmHg [80] would drive less than $0.9 \, \upmu \text {L/min}$ through the glymphatic circulation.

#### ECS

To estimate a lower bound for the resistance in the ECS, we used $\kappa = 20 \, {\text {nm}}^2$, the highest esimated permeability from Holter et al. [14], a viscosity $\mu = 0.7 \times 10^{-3} \, \text {Pa s}$ and a pressure difference of 1 mmHg between arteriole and venule. This pressure difference is arbitrary when the only output parameter of interest is the resistance to flow. The total flow Q in the ECS was computed according to Eq. (12), and the corresponding resistance was computed to be

27 $$\begin{aligned} R_{\text {ECS}} = \frac{Q}{\Delta p} = 0.57 {\text {mmHg/(mL/min)}}. \end{aligned}$$

The total resistance in the ECS is surprisingly low compared to the seemingly low permeability in the same space, and $R_{\text {ECS}}$ is of similar magnitude as $R_{\text {PVS}}$ as computed by Faghih and Sharp [11].

#### Inter-endfeet gaps

An IEG surface area of $0.005 \, {\text {m}}^2$ (0.3 % of artery/arteriole and vein/venule surface area), resulted in $n_a = 14.14$ and $n_v = 18.85$ IEG per cross section of arteriole and venule, respectively. Taking all $N_a = 125$ arterioles and $N_v = 50$ venules within the geometry, the total resistances were computed to be

28 $$\begin{aligned} R_{\text {IEG,a}} = \frac{12 \mu T}{h_a^3 D\, n_a N_a} = 0.57 \text { mmHg/(mL/min)} \end{aligned}$$

29 $$\begin{aligned} R_{\text {IEG,v}} =\frac{12 \mu T}{h_v^3 D \, n_v N_v} = 0.64\,{\text {mmHg/(mL/min)}}. \end{aligned}$$

Thus, the route first through arterial IEG, then through the ECS and finally out through venous IEG seems more likely than transportation along capillary gaps, assuming a gap size of 100 nm as reported by Bedussi et al. [31]. Including IEG, the resistance to flow from arterial PVS through ECS to venous PVS was thus $R_{\text {IEG,a}} + R_{\text {IEG,v}} + R_{\text {ECS}} = 1.69 \, \text {mmHg/(mL/min)}$. The resistance through the entire glymphatic system was computed to be $R_{\text {glymph}} = R_{\text {aPVS}} + R_{\text {V}} = 2.73 \, \text {mmHg/(mL/min)}$.

### Flow distribution between compartments

Figure 3 shows the results from a standard infusion test with a production rate of 0.33 mL/min and constant infusion at a rate of 1.5 mL/min. Baseline ICP was 10.99 mmHg, and peak ICP was 24.28 mmHg, as compared with 11.66 and 24.16 in the reference model (model 0). A steady state is reached after approximately 20 min infusion, when the flow rate is 99% of that at 30 min. Flow from the PVS to the SAS gradually decreases and becomes stagnant after 8 minutes of the infusion test. The outflow SAS resistance (as defined by Eq. (20) was $R_{\text {0}} = 8.67 \, \text {mmHg/(mL/min)}$. It should be noted that calculations of $R_{\text {0}}$ in Table 3 assumed a capillary flow linearly dependent on ICP, while in our model $R_{\text {0}}$ increased slightly when ICP rose above 20 mmHg. Nevertheless $R_{\text {0}}$ did not change considerably above 20 mmHg, and the reported values still provide a good estimate for the outflow resistance of the respective models. The full model showed very good agreement in resistance to outflow with the reference model and was well within values of $R_{\text {out}}$ as seen in healthy subjects [3]. Despite similar ICP, flow through (the only outflow route) the arachnoid granulations were greater in the reference model than in the full model.

At baseline ICP, flow through the AG was 0.24 mL/min, while 0.16 mL/min left the system through the cribriform plate. Net capillary filtration was 0.07 mL/min, distributed to the SAS along (arterial) PVS (0.04 mL/min) and paravenous spaces (0.03 mL/min, data not shown). The distribution of clearance from the SAS to each compartment was thus found to be 60.0% to AG, and 40.0% to the cribriform plate. Capillary filtration was responsible for 17.5% of CSF production, distributed to the SAS out along both arterial and venous PVS.

**Fig. 4:**
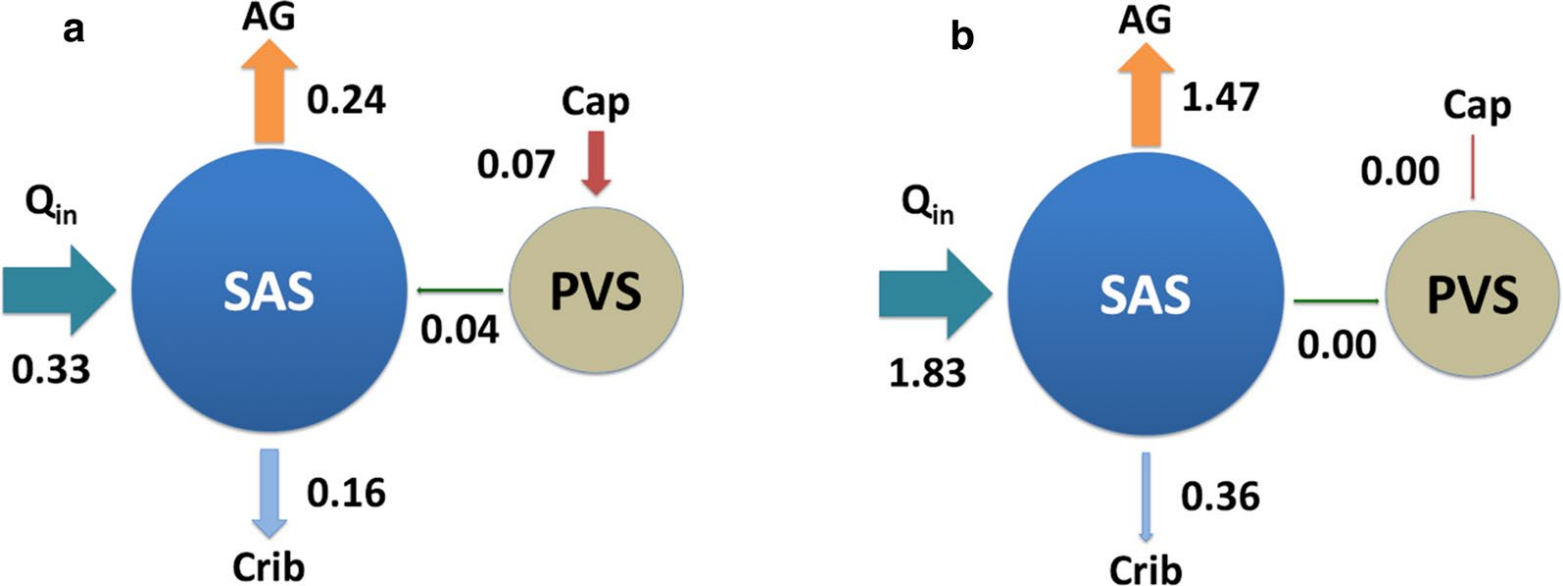
Illustration of the outflow distribution in mL/min **a** before and **b** during infusion. The size of the arrows are proportional to $Q_{{\text {in}}}$

At plateau ICP, clearance was also dominated by flow through the AG. In addition, capillary filtration stopped due to increased ICP, resulting in stagnant PVS flow. At plateau ICP, 1.47 mL/min was cleared through the granulations, while for the cribriform plate the flow rate was 0.36 mL/min. At plateau ICP the distribution of clearance from the SAS to the different compartments changed, with 80.3% exiting through the granulations, 19.7% into the cribriform plate, and no flow in PVS. Our main findings are also illustrated in Fig. 4.

### PVS velocity and pressure

At baseline, the PVS pressure was 11.02 mmHg as compared to 10.99 mmHg for the ICP (Fig. 3). During the infusion, ICP and PVS pressure became closer as ICP approached the capillary pressure. When ICP eventually exceeded the capillary pressure of 20 mmHg, capillary filtration ceased, and PVS pressure was equal to ICP.

Using Eq. (22), with $A_{\text {PVS}} = 38 \, {\text {cm}}^2$ and flow rate as given in Table 3, the resulting PVS velocities were $v_{\text {PVS}} = 0.18 \, \upmu \text {m/s}$ out to the SAS at baseline and no PVS flow during infusion.

### Model variations

The models stayed within the assumption of a plateau pressure below 26 mmHg (as discussed in “Methods” section), with the exception of models 5 and 6. The exact output values from these latter two models could thus be affected by the assumption of a pressure independent $R_{\text {out}}$. $R_0$, as defined in Eq. (20), denotes the total outflow resistance from the SAS compartment. $p_{\text {0,base}}$ is defined as baseline ICP, and is given as the first number under the ICP column. Instead of reporting the PVS pressure for all models, we rather report the PVS flow. The PVS pressure can be deduced from the PVS flow via Eq. (6) as $p_1 = p_0 + Q_{\text {aPVS}}R_{\text {aPVS}}$. Here, $Q_{\text {aPVS}}$ is the PVS flow as reported in Table 3, $p_0$ is the pressure in the SAS and $R_{\text {aPVS}}$ is the paravascular resistance.

#### Reference model (model 0)

The reference model behaved as expected, with a pressure increase from 11.66 to 24.16 mmHg during constant infusion at 1.5 mL/min. The plateau ICP in the SAS was at the transition phase for when the assumption of a pressure independent $R_{\text {out}}$ is valid ($\text {p} > 23{-}26 \, \text {mmHg}$).

#### Full model (model 1)

The results from the full model were described more in detail in the previous three sections. We note that the resistance to outflow from the SAS was almost identical in the full model compared to the reference model (Table 3, model 1 vs. model 0).

#### Direct glymphatic route to cervical lymphatics (model 2)

When paravenous spaces assumed to drain directly to cervical lymphatics, in a route separated from the SAS, the infusion test did not result in the expected increase of ICP. The ICP increased from 8.98 to 12.21 during the infusion. The lumped resistance to outflow from the SAS was only 2.16 mmHg/(mL/min), a value fivefold lower than the expected $R_{\text {out}}$. Due to the extremely low, $R_{\text {out}}$ for the system, resting ICP dropped, and AG flow decreased drastically compared to the full model. Flow over the cribriform plate and through PVS dominated at rest, while during infusion the PVS flow accounted for more than 2/3 of the outflow.

#### Constant capillary filtration (model 3)

Constant capillary filtration of water (flow rate independent of ICP), resulted in a constant PVS flow into the SAS. The (arterial) PVS flow rate was found to be 0.1 mL/min and thus the flow entering the SAS along venous PVS was 0.06 mL/min (the sum of arterial and venous PVS flow equals capillary filtration which was set to 0.16 mL/min). Similarly as for the full model, outflow through AG and cribriform plate were of comparable size (0.31 and 0.18 mL/min) at resting ICP, but AG dominated cribriform plate outflow at plateau ICP (1.61 vs. 0.38 mL/min). The ICP increased from 11.81 to 25.77 mmHg during infusion and thus had a similar pressure response to infusion as the full model.

#### Glymphatic pathway eliminated (model 4)

In this model, no flow was assumed in the PVS and the AG and the cribriform plate were assumed the only flow pathways, and capillaries did not filtrate net flow into the SAS. Due to the lower production/inflow of water to the SAS, ICP was lower at rest and flow through the cribriform plate was thus slightly lower in model 4 than in most of the other models. The AG and cribriform plate flow were similar at resting ICP (0.18 vs. 0.15 mL/min) while at higher ICP during infusion AG dominates cribriform plate flow (1.47 vs. 0.36 mL/min).

#### AG pathway eliminated (model 5)

In the special case where the AG pathway was eliminated, all fluid had to exit the system through the cribriform plate. The resulting ICP became high at rest (22.11 mmHg) and unphysiologically high (112.61 mmHg) during infusion. Due to the high ICP, capillaries did not filtrate water, neither at plateau nor resting ICP.

#### Increased AG resistance (model 6)

When the AG resistance was doubled, AG and cribriform plate flow were equal at resting ICP (0.20 vs 0.19 mL/min). During infusion the pressure increased from 12.71 to 36.28 mmHg, and at plateau ICP the AG flow again dominated cribriform plate flow (1.29 vs. 0.54 mL/min). Capillaries slowly filtrated water at rest (0.06 mL/min) while at plateau ICP net capillary filtration was 0.

#### Extended capillary gaps (model 7)

In model 7, it should be noted that we also assumed an outflow pathway from the glymphatic system directly to cervical lymph via pial sleeves. Without this assumption, the model would be almost identical to the full model, resistance to pressure driven flow from the capillaries into the SAS is dominated by resistance over the capillary wall, making a decrease in another (already small) serial resistance negligible. The cumulative resistance for the extended capillary gaps was found to be $0.9 \times 10^{-3} \, \text {mmHg/(mL/min)}$. With the assumptions described for model 7 in the methods section, the smallest capillary gaps was computed to be $1.2 \, \upmu \text {m}$, a result seemingly more in agreement with figures by Pizzo et al. [30] (in particular their Figure 9H). Following the steps from Faghih and Sharp [11], we calculated the resistance in the paraarterial tree, without the assumption of smaller gaps on the final generation in their model, to be $1.48 \times 10^{-3} \, \text {mmHg/(mL/min)}$. The latter calculation seems to be in good agreement with their presented figure of cumulative resistance in the paraarterial tree. With these resistances, the ICP was even less responsive to infusion than in model 2. The ICP increased from 8.42 to 8.43 mmHg during infusion, while PVS, and further flow to cervical lymphatics dominated the total outflow at 0.20 mL/min at rest and 1.70 mL/min at plateau ICP. The cribriform plate flow did not change during infusion, and was found to be 0.13 mL/min. Capillaries also filtrated water into the brain and directly into cervical lymph via pial sleeves at a constant rate of 0.13 mL/min. The PVS flow rate yields an average PVS velocity of $7.46 \, \upmu \text {m/s}$ according to Eq. (22).

## Discussion

In this work, we computed CSF resistance, pressure and outflow both during resting state and during infusion. The mathematical model extended the traditional infusion modeling with pathways related to the glymphatic system. In addition, we calculated PVS pressure, flow and average velocity in both states. Model 1, 3, 4, and 6 all gave reasonable ICP both at rest and during constant infusion of fluid at a rate of 1.5 mL/min. Model 2 and 7, which involved significant flow in the PVS, did not result in the expected ICP increase during infusion. On the other hand, model 5 eliminated AG flow and caused unphysiologically high pressures. The best match between the traditional reference model and the proposed model was achieved with the full model (model 1), but the model with constant capillary filtration (model 3) also showed good agreement in ICP for both baseline and plateau levels.

Our estimate of resistance along the capillary gaps was on the same order of magnitude as resistances to other outflow routes such as the cribriform plate and arachnoid granulations as measured by others [55, 56, 64]. However, the resistance in the capillary gaps was about 30 times greater than the resistance in the paraarterial tree used in our model, the latter based on estimations by Faghih and Sharp [11]. In particular, such an increase in resistance on the capillary level renders flow along capillary PVS unlikely. In our case, a reasonable flow rate would require a pressure drop of 4.19 mmHg. Such flow must rely on local ICP differences in the SAS, and would be expected to induce flow directly along the SAS rather than through the possibly high-resistant glymphatic system. In addition, pressure gradients in the SAS are likely less than 3 mmHg/m [81, 82], and the transmantle pressure difference has been estimated to be no more than 0.03 mmHg [80]. Glymphatic circulation driven by local differences in pulsatile pressure of several mmHg also seems implausible, as the ICP wave is almost synchronous throughout the brain [83]. Maximal estimated pulsatile pressure differences in the brain have been reported at no more than 0.2 mmHg [84], and a net driving force would be expected to be even lower.

In contrast to flow along the capillary gaps, resistance in the ECS was found to be surprisingly low at 0.57 mmHg/(mL/min), approximately half the resistance of that reported in arterial PVS by Faghih and Sharp [11]. The inclusion of IEG increased resistance with a factor 3 to yield a resistance of only 1.78 mmHg. Including PVS, the resistance increase to nearly 3 mmHg/(mL/min), and a pressure difference of 1 mmHg would thus have the potential to drive flow comparable to CSF production through the glymphatic circulation. To this end, we found a direct communication between the glymphatic circulation and cervical lymphatics unlikely. If the counter pressure at the level of cervical lymphatics stays relatively stable during infusion, an ICP increase of several mmHg is not possible as all infused CSF will exit with low resistance through the glymphatic circulation to cervical lymphatics.

Ma et al. [19] suggest that outflow through the cribriform plate dominate, but only when the total outflow from the SAS is large. We also found the relative distribution of flow to the different outflow pathways to be affected by infusion, but there are important differences. In contrast to Ma et al. [19], we found the AG to become even more important at high outflow rates (i.e. at higher infusion rates and high ICP). With the full model, at baseline ICP, AG flow was 50% greater than flow through the cribriform plate, and fourfold greater at plateau ICP. AG flow was greater than or equal to flow through the cribriform plate in all cases in all models except for models 2, 5 and 7, which were all models not able to predict the expected increase in ICP during the infusion test. Flow in PVS has been assessed under both awake and anaesthetized conditions. While we did not address this question, there is conflicting evidence whether PVS flow increases or decreases during sleep or anesthesia [19, 85, 86]. To what extent pressure, outflow resistances, or CSF production cause changes between different states is not well understood.

Net capillary flow in our model was always directed from the capillaries to the PVS. Thus, under normal conditions, the capillaries functioned as a site of CSF production. This suggests net fluid movement from the PVS into the SAS in the framework of a circular glymphatic system. In models we tested where pressure exceeded 20 mmHg, the capillaries ceased net filtration. It is interesting to note that this pressure threshold of above 20 mmHg is close to the value for which $R_{\text {out}}$ becomes pressure dependent as measured experimentally [39]. Capillary flow rates were relatively small, up to 0.16 mL/min when a constant flow was assumed (model 3), and less than 0.1 mL/min when the flow was modelled. Capillaries functioning as a route of absorption and filtration of CSF is in line with the Bulat-Klarica-Orešković hypothesis [21]. However, it should be noted that passage of substances such as proteins and electrolytes is difficult across the blood–brain barrier as compared to water [87]. The routes of CSF/water clearance (CSF is 99% water [21]) do not necessarily align perfectly with the routes of clearance of other substances from the brain. We finally note that our estimation of capillary resistance is one order of magnitude lower than what has been estimated by Koch [88].

The average PVS velocity in the full model was $0.18 \, \upmu \text {m/s}$ out from the PVS into the SAS at baseline and no flow occurred at plateau ICP. The maximal recorded PVS velocity was computed to be $7.46 \, \upmu \text {m/s}$, during infusion in model 7, in which almost all infused and produced CSF went through PVS. In the experimental studies, several investigators used an infusion rate of $2 \, \upmu \text {L/min}$ in rodents [6, 8, 29], which was shown to increase ICP by 2.5 mmHg [6], a substantial increase, but less than in our model of a human with an infusion rate of 1.5 mL/min. In addition, Bedussi et al. [7], used a much lower infusion rate of $0.34 \, \upmu \text {L/min}$, only resulting in a pressure rise of 0.1 mmHg. Still, the net PVS velocity of $17 \, \upmu \text {m/s}$ found with this low increase in ICP is almost identical to the typical flow speed of $18.7 \, \upmu \text {m/s}$ found by Mestre et al. [8] at the infusion rate of $2 \, \upmu \text {L/min}$, suggesting elevated ICP is not the sole reason for PVS flow. It should be noted that both Bedussi et al. [7], and Mestre et al. [8] consider PVS at the brain surface. All observed CSF flowing in these spaces does not necessarily need to follow PVS into the brain, but could also be drained to other outflow pathways. Thus, increased AG or cribriform plate flow during infusion in our model could very well be in accordance with increased velocities around arteries on the brain surface.

Our findings suggest that average velocities up to $20 \, \upmu \text {m/s}$ are unlikely around penetrating arteries in the parenchyma of the human brain as the corresponding flow rate in all PVS combined would be threefold greater than the CSF production rate and the infusion rate combined. It should be noted that our model applies to humans, while the experimental findings [7, 8] concern rodents. Even if the infusion rate is scaled with CSF volume of the species, the mouse ICP could be expected to change less because CSF turnover time is $\approx 3$ times shorter in mice than in humans [9]. In addition, a shorter turnover time may also suggest higher velocities related to drainage of CSF in mice.

The current study gives new insight to the relation between ICP and CSF clearance. This is not only highly relevant to the glymphatic theory, but also to understand pathological conditions related to increased ICP. Patients with increased ICP often exhibit clinical visual symptoms, which are also typical in idiopathic intracranial hypertension (IIH) and in astronauts suffering from Spaceflight Associated Neuro-ocular Syndrome (SANS) [89].

*Limitations*

Resistance parameters in our model were taken from many different species and types of experiments. Ideally, resistance in each outflow pathway could be measured experimentally by blocking one outflow pathway, and measuring the resulting increase in pressure. To our knowledge, this has not been done in humans and it is likely challenging both from a technical and ethical perspective. Specifically, the exact size of capillary and inter-endfeet gaps for CSF/ISF flow would provide answers to whether a circular glymphatic system is plausible. The current data suggest a resistance of 32.24 mmHg/(mL/min) to flow along the capillary gaps, while increasing the gap radius reduces this resistance with several orders of magnitude. In addition, more data on what happens at the venous side of the glymphatic system (e.g. whether PVS form a route directly to cervical lymphatics or a return to the SAS, the magnitude of the potential counter pressure at cervical lymphatics etc.) would increase the robustness of the model presented. Thus, the finding of a low resistance pathway through from PVSa-ECS-PVSv was crucial for disproving a glymphatic pathway with drainage through pial sleeves to cervical lymphatics. An approximately fifteen times increase in resistance would render a PVSa-ECS-PVSv flow in balance with AG flow and accordingly both give a relevant glymphatic pathway and a total outflow resistance that are in agreement with pressure increases seen in typical infusion tests. This emphasizes the importance of additional experimental research to investigate these critical resistances. Although our model well describes the time-evolution of the CSF pressure in the SAS during infusion test, additional experimental data may further allow for detailed dynamic response of pressure in the other compartments shown in Fig. 1, such as e.g. the ECS.

Our model did not include the effect of cardiac or respiratory pulsatility. Pulsations in the arterial, venous and CSF compartments have all been proposed to drive glymphatic clearance [6–8]. Indeed, the cardiac pulsation on the arterial side seems related to the PVS pulsatile movement as shown in Mestre et al. [8], but modeling attempts deem it unlikely that arterial wall movements alone drive a net flow of sufficient magnitude for clearance of fluid [16, 54]. In addition, when ignoring pulsatility, the large inflow of blood on the arterial side during systole will not affect the compliance of the system. Adding pulsatility to the presented linear compartment model would not change the average distribution of flow rates to the different compartments. A valve-like mechanism could be modeled by assuming the PVS resistance to be different in the two directions. However, at present no such valves have been identified [90].

According to Tithof et al. [91], PVS are not concentric cylinders, but rather form ellipses around vessels to minimize resistance. This geometrical change along all vessels may decrease resistance by a factor of 2–3 [91], and thus likely increase PVS velocities by a similar factor in some of our models. In addition, peak velocity in a concentric cylinder is double that of the mean velocity, which possibly may increase our velocity estimates in some of the models by another factor of approximately two.

In the current study we only consider supine position and thereby we neglect hydrostatic effects [61, 92]. This simplification justifies the representation of a single compliance for the craniospinal space and allows us to lump spinal absorption into the AGs [28]. Spinal absorption is believed to increase in upright posture due to hydrostatic effects [60, 92]. Including spinal arachnoid villi in the AG resistance would reduce the magnitude of this parameter approximately by 20% [58–60]. This will not change our conclusions from the model, and if anything it will make AG even more important in terms of total outflow.

A single craniospinal compliance assigns all compliance to the SAS. However, for transient analysis, a compliance distributed between the physiological units including the SAS and adjacent compartments could be more appropriate. In this latter case, quantification of the separate compartment compliances would be needed. Further, in this study, we have not accounted for spatial variations, but rather assumed a compartment model in which the pressures are functions of time only. We note that ICP has been shown to be nearly constant in space [83], whilst the blood flow pulse propagation has been reported to show spatial directionality [93, 94].

Capillary filtration is regulated by osmotic gradients [21, 23] and cotransporting proteins [76], which were not considered in our study. The effective capillary pressure was assumed constant in time for $p_1 < p_{\mathrm{cap}}$, for higher PVS pressure the capillary filtration ceased and $p_{\mathrm{cap}}$ was set equal to $p_1$. Whether the capillary pressure always stays above ICP regardless of ICP increase is not well known. In model 3, we ensured that a pressure independent constant filtration model (and also alterations in the magnitude of filtration) yielded reasonable results. A constant filtration over the blood–brain barrier is only possible if we allow a flux of solutes, while in the current study we have not computed ion permeabilities and solute fluxes across the blood–brain barrier.

## Conclusions

According to our models of CSF clearance, outflow predominantly occurred through the arachnoid granulations, both during baseline and plateau ICP. The glymphatic resistance was found too high to suggest a circular glymphatic system, but at the same time too low to suggest a direct route from venous PVS to cervical lymphatics. Paravascular flow occurred from the PVS to the SAS at baseline ICP, due to capillary filtration, and was stagnant at plateau. We conclude that ICP increase is an important factor to address when determining the pathways of injected substances in the SAS.

## Supplementary information

**Additional file 1.** Additional figure showing computational 2D model of ECS flow between arteriole PVS to venous PVS. Top: The entire computational domain and the computed pressure field. Arteriole PVS are surrounded by regions of high pressure (red) while venule PVS are surrounded by regions of low pressure (blue). Bottom: Closer look at a section of the top panel, with directions of the corresponding flow field superimposed on the pressure field. Flow occurs from arteriole to venule PVS. Arrow size indicate the magnitude of the flow.

**Additional file 2.** Parameters from the 2D model used to compute the total resistance in the extracellular space andthrough inter-endfeet gaps both on the arterial and venous side.

## Data Availability

No original datasets were used in the present study. All parameters used in the model are described in the methods section and illustrated in the additional figures.
