# Supplementary figures and images for: Intracranial pressure elevation alters CSF clearance pathways
Source: Fluids Barriers CNS. 2020 Apr 16;17:29. doi: 10.1186/s12987-020-00189-1 (PMC7161287; doi:10.1186/s12987-020-00189-1)

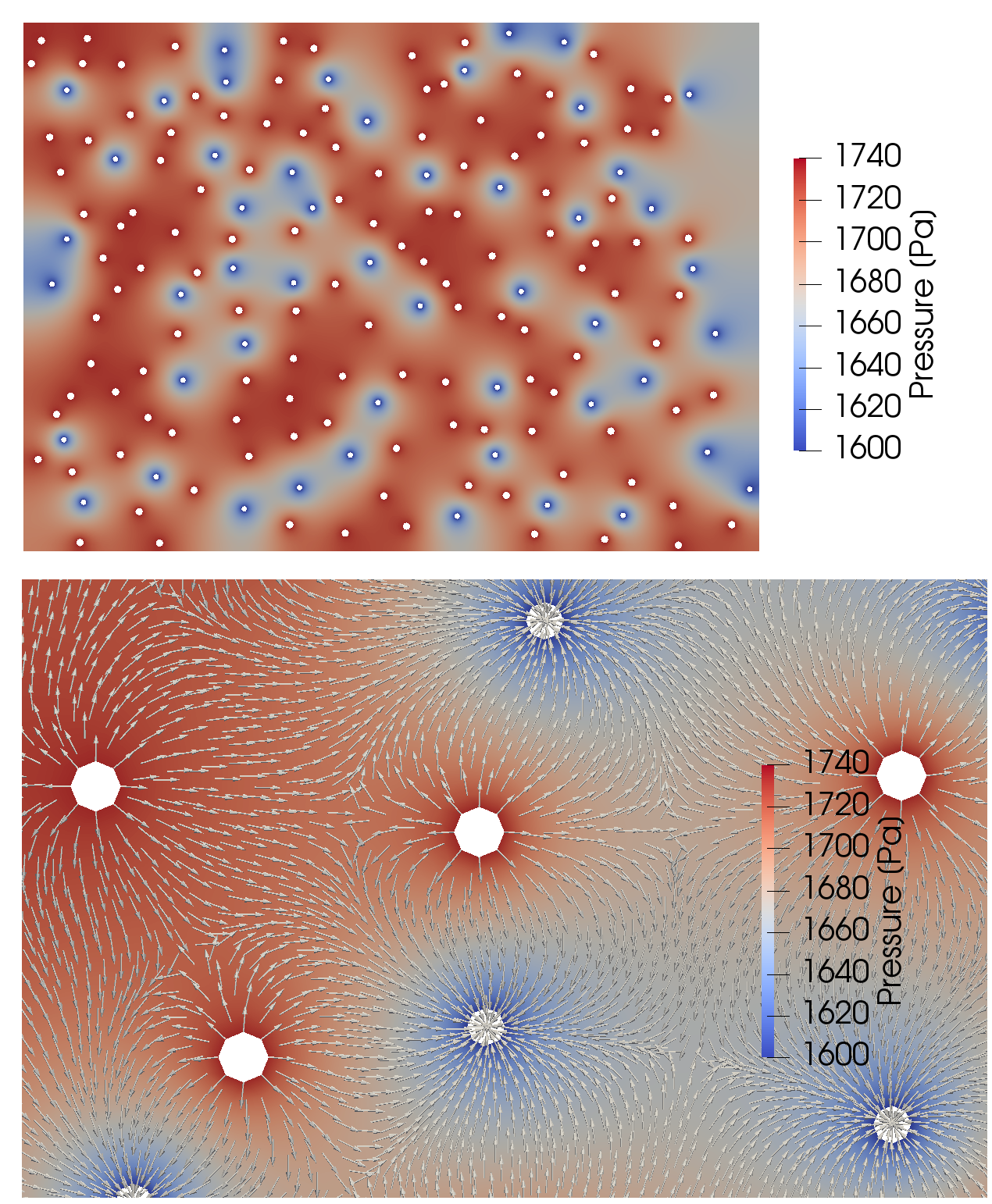

Supplement: Supplementary file 1 — Additional file 1. Additional figure showing computational 2D model of ECS flow between arteriole PVS to venous PVS. Top: The entire computational domain and the computed pressure field. Arteriole PVS are surrounded by regions of high pressure (red) while venule PVS are surrounded by regions of low pressure (blue). Bottom: Closer look at a section of the top panel, with directions of the corresponding flow field superimposed on the pressure field. Flow occurs from arteriole to venule PVS. Arrow size indicate the magnitude of the flow. [file 12987_2020_189_MOESM1_ESM.png]
