# Supplementary material for: Intracranial pressure elevation alters CSF clearance pathways
Source: Fluids Barriers CNS. 2020 Apr 16;17:29. doi: 10.1186/s12987-020-00189-1 (PMC7161287; doi:10.1186/s12987-020-00189-1)
Supplement: Supplementary file 2 — Additional file 2. Parameters from the 2D model used to compute the total resistance in the extracellular space andthrough inter-endfeet gaps both on the arterial and venous side. [file 12987_2020_189_MOESM2_ESM.pdf]

Additional file 2. Parameters from the 2D model used to compute the total resistance in the extracellular space and through inter-endfeet gaps both on the arterial and venous side.

Parameter	Value
Computational domain of 2D ECS	$L = 3.91$ mm, $H = 2.81$ mm
Number of arterioles and venules in 2D domain	$N_a = 125$, $N_v = 50$
Arteriole and venule radius	$r_a = 15\mu$m, $r_v = 20\mu$m
Endfeet thickness	T $= 1$ μm
Inter-endfeet cleft gaps	$h_a = 24$ nm, $h_v = 31$ nm [52, 53]
Artery/arteriole and vein/venule area	$A = 1.64$m^2
Total inter-edfeet cleft gap area	$A_{\text{EF}} = 0.003A \approx 0.005$ m ^{2} [53]
Inter-edfeet cleft length along entire vessel	$D = 90.67$ m
Average number of clefts per vessel	$n_a = 14.14, n_v = 18.85$
